# ALNet: towards real-time and accurate maize row detection via anchor-line network

**DOI:** 10.3389/fpls.2025.1706596

**Published:** 2025-12-01

**Authors:** Bofeng Feng, Qingliang He, Yun Hu, Hao Cai, Dongyu Luo, Zhiye Shen, Bob Zhang, Long Qi, Ruijun Ma

**Affiliations:** 1 College of Engineering, South China Agricultural University, Guangzhou, China; 2 School of Traffic and Transportation, Beijing Jiaotong University, Beijing, China; 3 Department of Computer and Information Science, University of Macau, Macao, Macao SAR, China; 4 College of Water Conservancy and Civil Engineering, South China Agricultural University, Guangzhou, China; 5 Guangdong Engineering Technology Research Center of Rice Transplanting Mechanical Equipment, Guangzhou, China; 6 State Key Laboratory of Agricultural Equipment Technology, Guangzhou, China; 7 Department of Biosystems Engineering, University of Manitoba, Winnipeg, MB, Canada

**Keywords:** corn row detection, anchor-line, attention-guided ROI align, dual-axis extrusion transformer, precision agriculture

## Abstract

Accurate and efficient crop row detection is essential for the visual navigation of agricultural machinery. However, existing deep learning–based methods often suffer from high computational costs, limited deployment capability on edge devices, and difficulty in maintaining both accuracy and speed. This study presents ALNet (Anchor-Line Network), a lightweight convolutional neural network tailored to the elongated geometry of maize rows. ALNet introduces an Anchor-Line mechanism to reformulate row detection as an end-to-end regression task, replacing pixel-wise convolutions with row-aligned kernel operations to reduce computation while preserving geometric continuity. An Attention-guided ROI Align module equipped with a Dual-Axis Extrusion Transformer (DAE-Former) is incorporated to capture global–local feature interactions and enhance robustness under challenging field conditions such as weed infestation, low light, and wind distortion. In addition, a Row IoU (RIoU) loss is designed to improve localization accuracy by aligning predicted and ground-truth row geometries more effectively. Experimental results on field-acquired maize datasets demonstrate that ALNet achieves an *mF*1 of 59.60 across IoU thresholds (≥ 9.24 points higher than competing methods) and an inference speed of 161.26 FPS, with a computational cost of only 11.9 GFlops, demonstrating potential for real-time edge deployment. These advances establish ALNet as a practical and scalable solution for intelligent visual navigation in precision agriculture.

## 1 Introduction

### 1.1 Background

Corn, the largest global crop and a primary staple in China, demands highly effective field management to ensure sustainable production Yao et al. (2025). Precision management practices are critical for creating optimal growth conditions, mitigating losses from diseases, pests, and weeds, and improving both yield and quality while reducing operational costs ShaoKun (2017); Zhaosheng (2025). In recent years, machine vision systems have become pivotal tools in modern agriculture, enabling advanced perceptual capabilities for autonomous field operations Abbasi et al. (2022); Rabab et al. (2021).

Among these applications, corn row detection stands as a foundational visual perception task, directly influencing the efficiency and accuracy of agricultural machinery in field management Chen et al. (2025); Diao et al. (2023). However, achieving reliable corn row detection in real-world scenarios remains challenging due to several factors: (1) Illumination variability: Variable lighting, shadows, and insufficient illumination can degrade image quality. (2) Weed interference: Dense weed growth with similar visual characteristics to corn can cause misidentification Chen et al. (2025). (3) Seedling variation: Missing plants Xiangguang (2021) and seedlings at different growth stages reduce detection accuracy. (4) Image blur: Machinery vibration can blur images, impairing detection performance. (5) Wind effects: Strong winds can temporarily lodge corn plants, distorting their visual features.

### 1.2 Related work

Existing deep learning-based crop row detection methods can be categorized into three types based on the algorithm used: object detection-based methods, semantic segmentation-based methods, and instance segmentation-based methods.

#### 1.2.1 Object detection-based methods

Current corn row detection methodologies typically employ a two-stage approach: plant localization followed by row aggregation. Hongbo (2024) developed a YOLOv8-G variant for corn seedling center detection, implementing affinity propagation clustering and least squares regression for row fitting. Lulu (2024) combined Faster R-CNN with Susan operator feature extraction, utilizing RANSAC algorithms to enhance row fitting precision. Gong and Zhuang (2024) improved YOLOv7-tiny through infrared imaging integration and ShuffleNet v1 optimization, incorporating Coordinate Attention mechanisms and EIOU loss functions to boost localization accuracy. While these approaches demonstrate technical merit, three fundamental limitations persist: (1) overhead imaging perspectives constrain practical field applicability, (2) computational complexity limits deployment on resource-constrained devices, and (3) processing speeds face challenges in meeting real-time agricultural operation requirements.

#### 1.2.2 Semantic segmentation–based methods

Semantic segmentation approaches offer streamlined solutions for crop row detection by directly predicting row structures from pixel-level classifications. Liu et al. (2024) developed a canopy ROI segmentation method coupled with horizontal striping analysis and midpoint clustering for row extraction. Cao et al. (2022) enhanced the ENet architecture through residual connections, implementing an optimized RANSAC algorithm for precise navigation path identification. Ban et al. (2025) combined StemFormer-based segmentation with LiDAR point cloud processing to cluster maize stalk positions and fit row trajectories. Gan et al. (2025) demonstrated the effectiveness of semi-supervised learning through their Unimatch framework, achieving efficient rice seedling segmentation with reduced annotation requirements. While these end-to-end methods eliminate multi-stage processing bottlenecks, they face three persistent challenges: (1) blurred edge delineation in dense canopies, (2) feature confusion in overlapping plant regions, and (3) contextual information loss during high-resolution processing Ronneberger et al. (2015); Takikawa et al. (2019). These limitations ultimately constrain detection accuracy under complex field conditions.

#### 1.2.3 Instance segmentation–based methods

Unlike semantic segmentation, methods based on instance segmentation typically employ a two-stage approach. For instance, they first identify the region where the instances are located, and then perform semantic segmentation within the detected bounding box, outputting each segmentation result as a separate instance. Wei et al. (2022) developed the RASCM model, reformulating row detection as optimal line-position selection through reinforced attention mechanisms. Bing (2021) proposed a perspective transformation pipeline that converts field images to bird’s-eye views before segmenting row-parallelogram masks. Chen et al. (2025) designed a dual-path network architecture that concurrently performs semantic segmentation and feature embedding, enabling simultaneous rice row clustering and trajectory regression. Although such methods offer finer granularity and improved speed over pure semantic segmentation, they still face challenges:(1) Contextual fragmentation: Treating crop rows as isolated entities disregards field-scale spatial relationships. (2)Computational intensity: Dense pixel-wise predictions require 1.8-3.2× more operations than detection-only methods. (3)Accuracy-speed tradeoff: Current implementations struggle to maintain 70% mAP while achieving 100ms latency.

### 1.3 Limitations and motivation

Despite considerable progress, most deep learning–based crop row detection methods face four major limitations: (1) Loss of holistic context–Treating crops and rows as independent objects reduces accuracy. (2) High computational cost–Pixel-wise prediction is expensive and slows inference. (3) Poor deployability–Heavy models consume excessive memory and are unsuitable for edge devices. (4) Speed–accuracy trade-off–Achieving both remains a challenge for field-scale application.

To address these issues, we propose ALNet (Anchor-Line Network), a deep learning corn row detection network based on an Anchor-Line strategy. This approach departs from conventional pixel-wise convolution by aligning convolutional kernels along the row direction, thereby lowering computational costs. A multi-scale feature extraction module combines deep semantic and shallow spatial cues, improving accuracy, while an Attention-guided ROI Align module recovers global context lost during Anchor-Line convolution. The network regresses entire maize rows as unified targets through a dedicated loss function.

The main contributions of this work are as follows:

- Anchor-Line design: tailored to maize row geometry, enabling end-to-end row extraction.
- Holistic target modeling: entire maize rows are detected and regressed as single entities.
- Edge deployment potential: a computationally efficient framework showing potential for edge device integration.
- Balanced performance: achieves both high accuracy and fast inference for practical production needs.

## 2 Materials and methods

**Figure 1:**
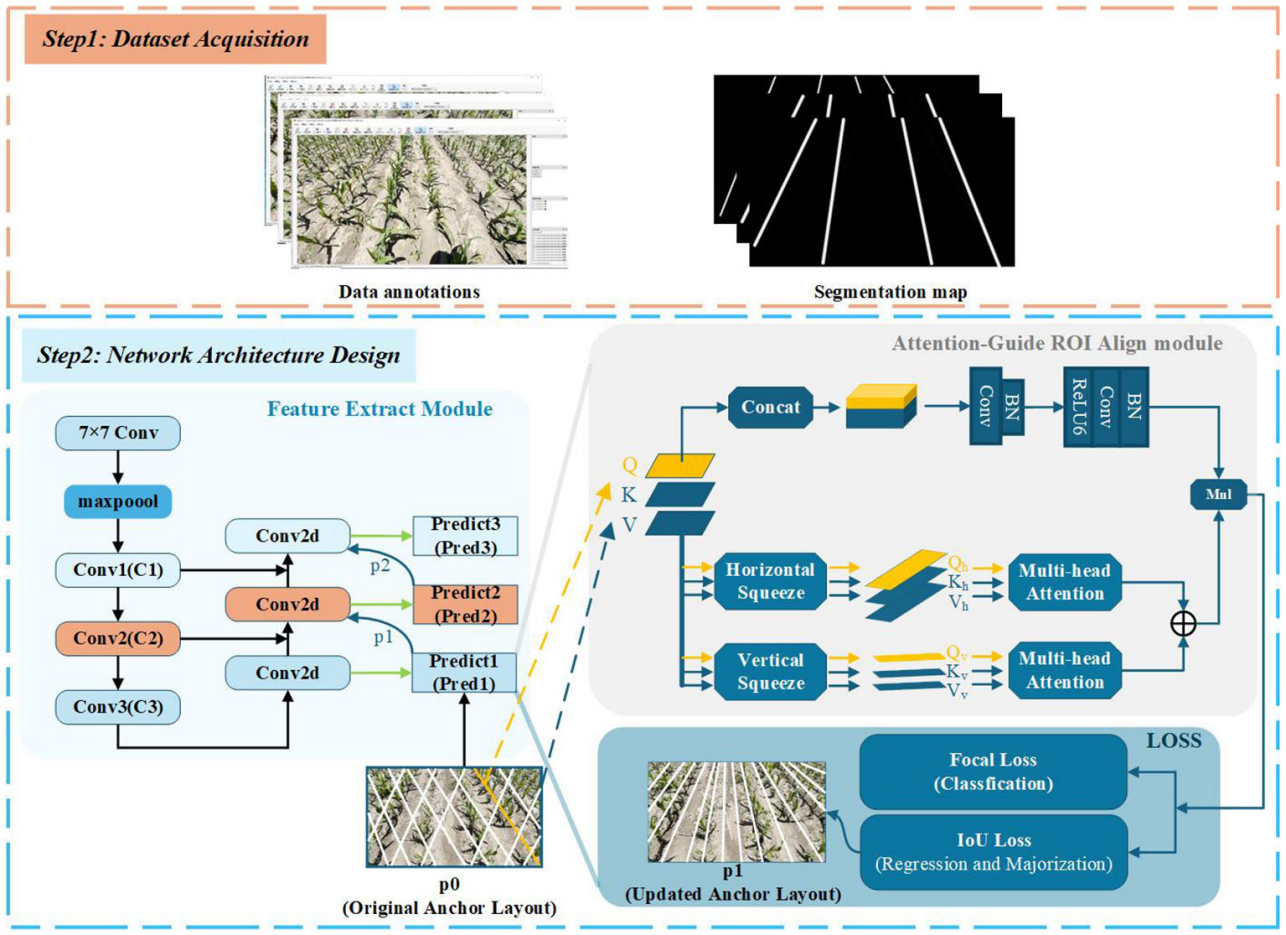
Flow diagram of the proposed crop row detection method.

As shown in **Figure 1** the general workflow of our method mainly includes two steps: First, maize images at the 3–5 leaf stage with unfolded leaves are collected and annotated to create a dataset for model training and testing. Second, the dataset is used to train the proposed network. Based on the learned features, the distribution of Anchor-Lines is optimized according to the shape and positional characteristics of maize rows, and an end-to-end detection model is established.

### 2.1 Dataset acquisition

**Figure 2:**
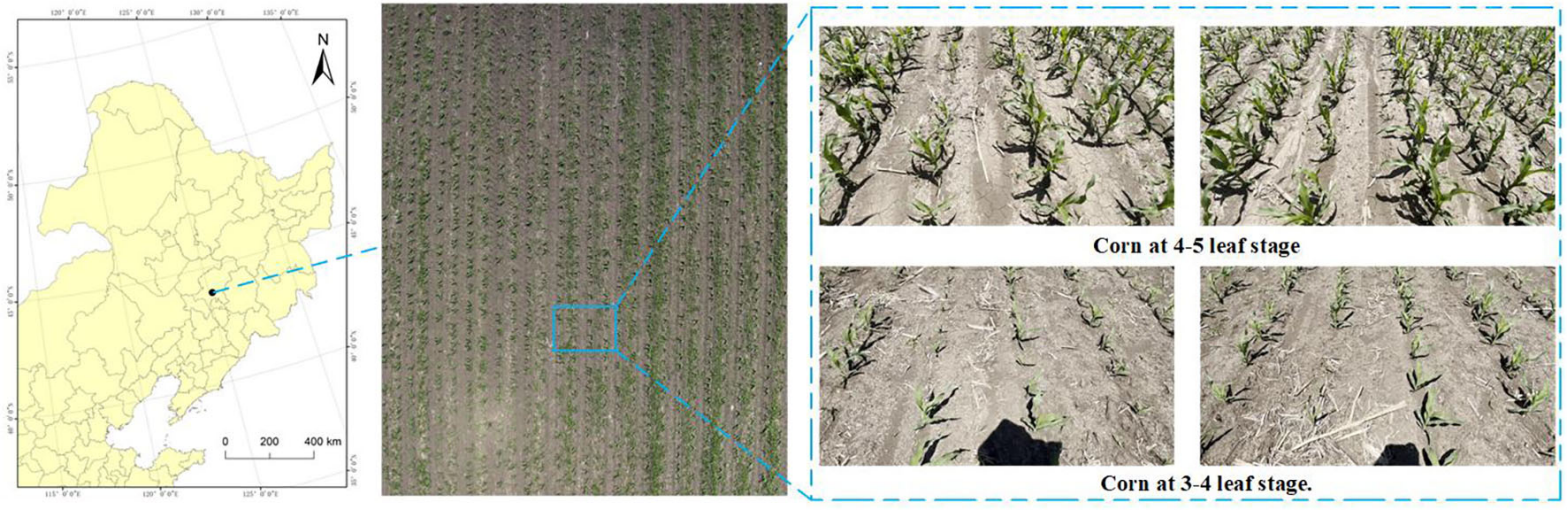
Data collection locations and sample images of corn rows.

As shown in **Figure 2**, the image data used in this study were collected from mechanically sown maize fields at the Jilin University Agricultural Experiment Base, located in Lvyuan District(43^°^49.02’N, 125^°^24.39’E), Changchun City, Jilin Province. Data collection was conducted in June 2024 and June 2025, when the maize was at the 3- to 5-leaf stage. At this growth stage maize roots begin to develop and weeds start to emerge. Maize leaves secrete substantial amounts of corn ketone, which can effectively reduce the toxicity of herbicides. Meanwhile, weeds are typically at the 2- to 4-leaf stage, during which their physiological activity is high and cell division is vigorous, rendering them more susceptible to herbicides. The efficiency of herbicide absorption and translocation is also higher at this stage, enabling more effective weed suppression Agathokleous (2018); Bing (2021).

**Figure 3:**
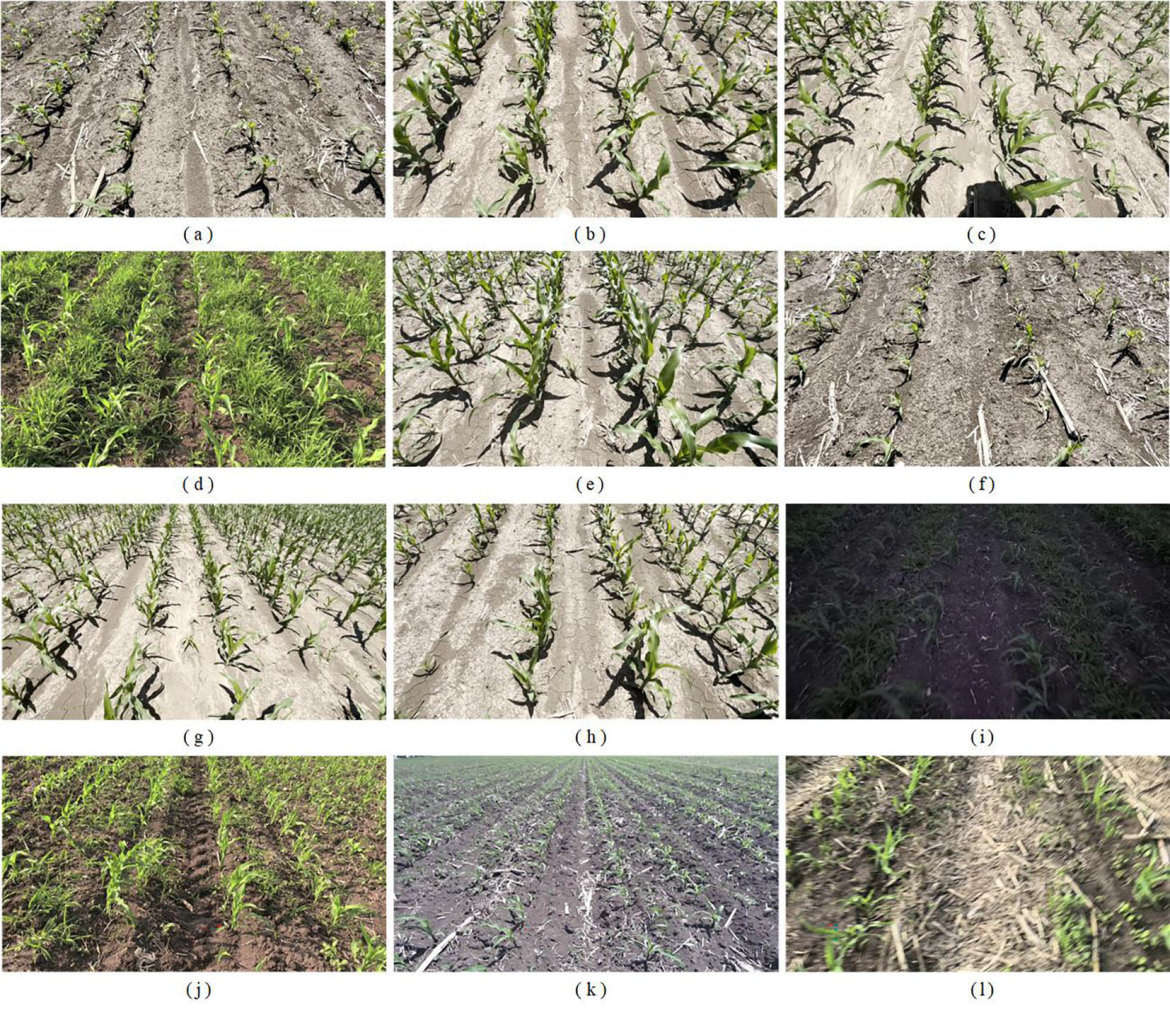
Some representative sample images of corn rows at the 3–5 leaf stage, including: **(a)** 3–4 leaf stage; **(b)** 4–5 leaf stage; **(c)** front-lit perspective; **(d)** weed-infested conditions; **(e)** imaging angle of 45^°^; **(f)** imaging angle of 60^°^; **(g)** imaging angle of 30^°^; **(h)** seedling gaps; **(i)** low-light conditions; **(j)** high-light conditions; **(k)** low-pixel images; **(l)** blurred images.

The images were acquired with an RGB camera mounted on a handheld gimbal, producing frames at a resolution of 1280×720. To facilitate practical deployment of the algorithm, three shooting angles were selected: 60^°^, 45^°^, and 30^°^; here the angle denotes the inclination between the camera optical axis and the horizontal plane. In total, 6000 maize images were recorded. The collected images cover various maize growth states and a wide range of complex field conditions, including dense weed coverage, missing seedlings, strong and low lighting, motion blur caused by agricultural machinery, and temporary lodging of maize due to strong winds. Several representative samples are shown in **Figure 3**.

All images were subsequently annotated using the open-source tool “Labelme”. Because the entire maize row is treated as the recognition target, annotations were made with polylines that trace each maize row.

### 2.2 Architecture overview

**Figure 4:**
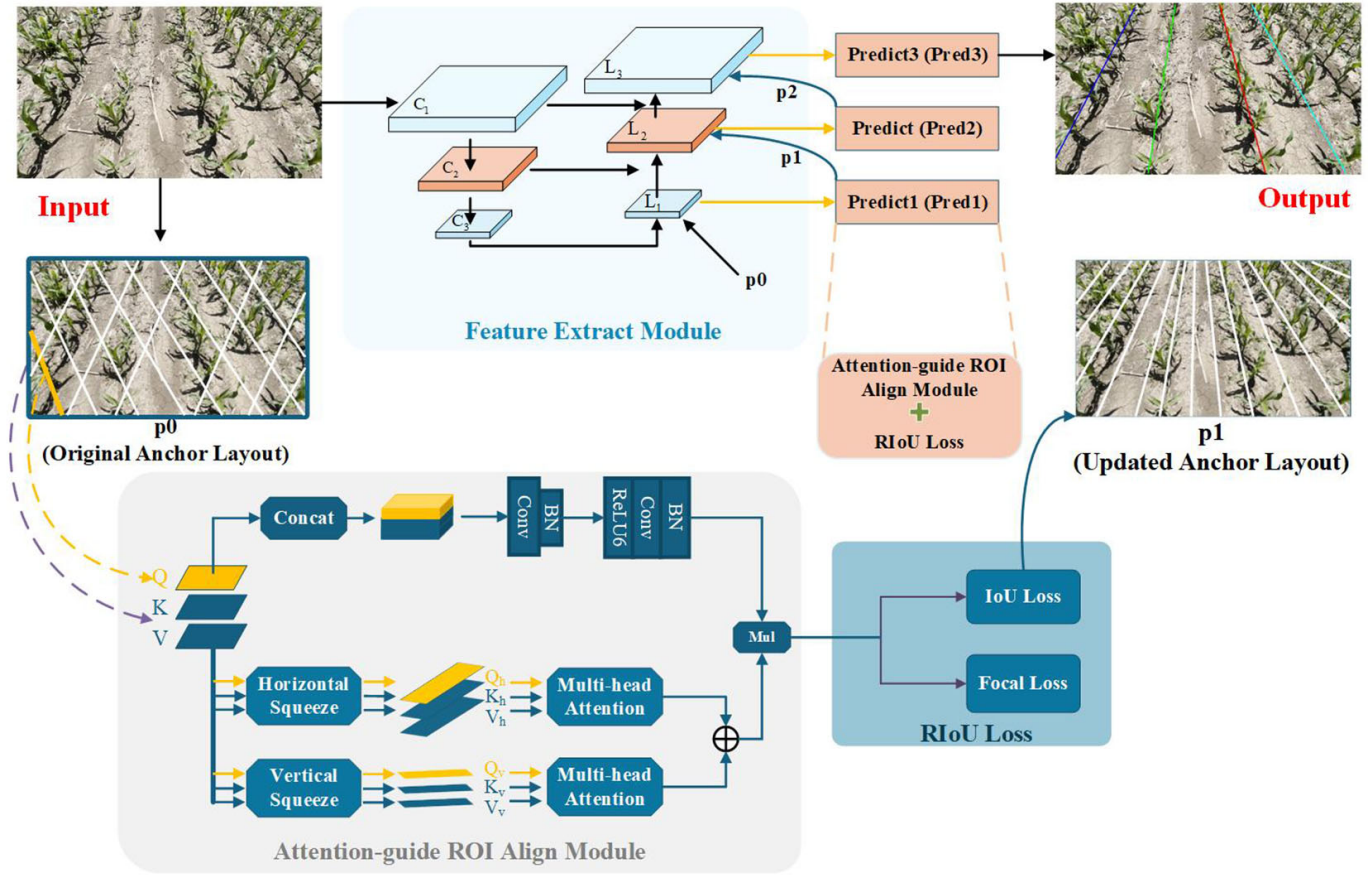
Overview of the proposed ALNet.

An overview of ALNet is depicted in **Figure 4**. The network mainly consists of three components: the Feature Extract module, the Attention-guide ROI Align module, and the RIoU loss. Specifically, the network accepts RGB images and outputs lines in different colors, each corresponding to a distinct maize row. As noted above, we treat the entire maize row as the detection target. In conventional object detection, rectangular bounding boxes are commonly used to represent targets Liu et al. (2016); Redmon et al. (2016); Ren et al. (2015); however, elongated maize rows are poorly represented by bounding boxes. Taking into account the prior knowledge of corn row shapes, we adopt an Anchor-Line based formulation that casts maize-row detection as a combined classification and localization problem. An end-to-end network architecture is thus designed to achieve efficient and accurate row detection. In the following subsections we describe each component in detail.

#### 2.2.1 Feature extract module

The Feature Extract module is responsible for extracting both deep semantic and shallow positional information from the input image, and for enabling flexible positioning of Anchor-Lines. Both accurate feature extraction and proper Anchor-Line placement are critical for high detection accuracy. To this end, we employed a Feature Pyramid Network (FPN) for multi-scale feature fusion He et al. (2016). Meanwhile, the detection head incorporated the Attention-guide ROI Align module and the RIoU loss (described below) to iteratively refine Anchor-Line positions so that they progressively aligned with the ground-truth maize rows. To mitigate gradient explosion and keep the model lightweight, ResNet-18 is adopted as the backbone Lin et al. (2017).

The specific workflow of the Feature Extract module is as follows. First, three convolutional layers are applied to the input image to extract multi-scale feature maps. The top-level feature map, C_3_, is passed through convolutional layer L_1_. An initial set of Anchor-Lines (p0) is introduced, and a convolutional kernel is applied sequentially along each Anchor-Line. The resulting feature maps are forwarded to the detection head Pred1. After Pred1 processes these features, an updated Anchor-Line arrangement (p1), which provides a more accurate estimate of the true maize row locations, is sent to L_2_. Concurrently, feature map C_2_ is fused with the convolved C_3_ (from L_1_), and this fused feature set is input to L_2_. In L_2_ the same convolution operation is performed along each Anchor-Line in p1. Finally, using the twice-updated Anchor-Line arrangement (p2) from L_2_, convolution is performed on the multi-scale fused feature map in L_3_; the result is fed into Pred3, which regresses the maize row lines.

#### 2.2.2 Attention-guide ROI align module

In practical field conditions, maize rows may be occluded or blurred due to complex scenarios such as extreme lighting, numerous weeds, or missing seedlings. Under such circumstances local visual cues are crucial to verify the presence of maize rows; therefore, relying solely on contextual information extracted by the FPN can be insufficient Lamping et al. (2025). To improve robustness, we propose the Attention-guide ROI Align module to better exploit long-range dependencies and aggregate richer contextual information for learning maize-row features. Concretely, convolutional operations aligned with the preceding Anchor-Line are added so that each pixel along an Anchor-Line can aggregate information from its neighborhood and thus enhance the representation of occluded or blurred regions. In addition, correlations between local features and the global feature map are established, allowing richer context to augment local feature representations and improve discrimination under challenging conditions. To ensure the Attention-guide ROI Align module remains lightweight enough for edge-device deployment, we replace the standard Vision Transformer attention Dosovitskiy et al. (2020); Liang et al. (2025a); Xie et al. (2021); Zheng et al. (2021) with the Dual-Axis Extrusion Transformer (DAE-Former). The implementation is as follows:

(1) $$\begin{array}{ll} q_{\left(h\right)} & =\frac{1}{W}{\left(q^{\to (C_{qk},H,W)}I_{W}\right)}^{\to (H,C_{qk})},\\ q_{\left(v\right)} & =\frac{1}{H}{\left(q^{\to (C_{qk},W,H)}I_{H}\right)}^{\to (W,C_{qk})}. \end{array}$$

**Figure 5:**
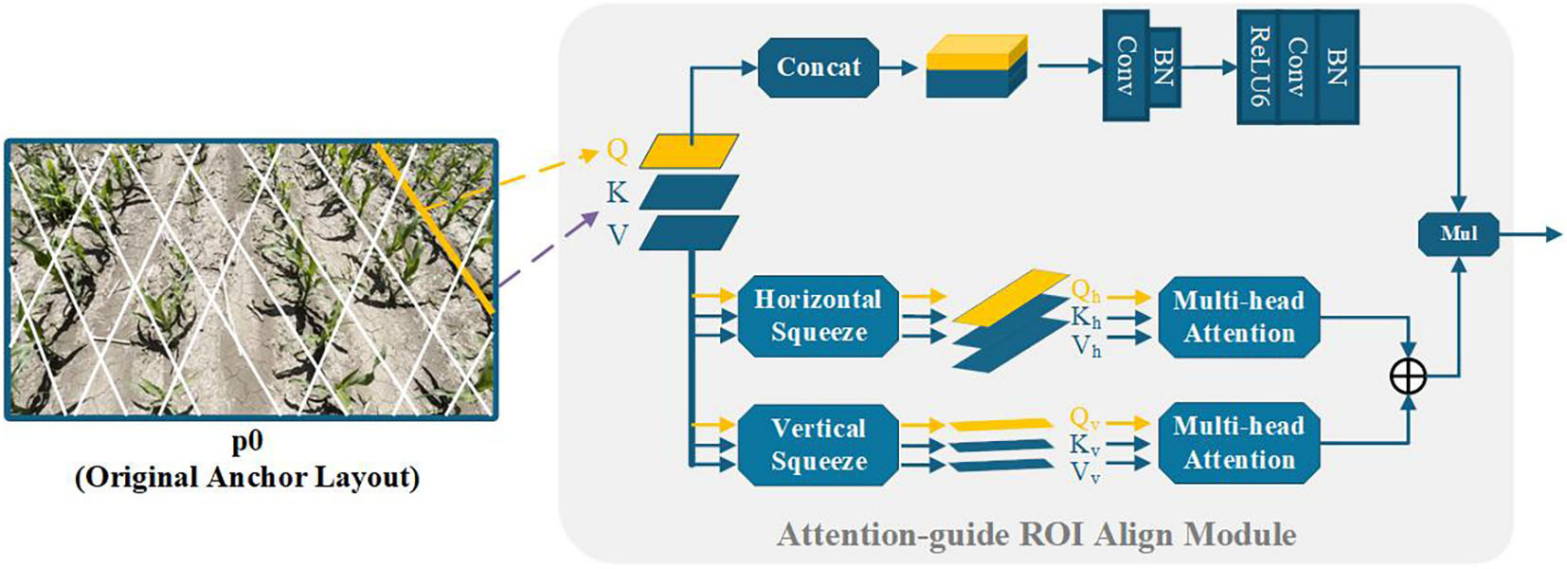
Overview of the attention-guide ROI align module.

As illustrated in the lower half of the Attention-guide ROI Align module in **Figure 5**, the query vector q is extracted from a single Anchor Line using a weight matrix $W_{q}^{\left(s\right)}\in {\mathbb{R}}^{C_{qk}\times C}$, while the key k and the value v are extracted from the future map using a weight matrix $W_{k}^{\left(s\right)}\in {\mathbb{R}}^{C_{qk}\times C}$, $W_{v}^{\left(s\right)}\in {\mathbb{R}}^{C_{v}\times C}$. According to the upper formula in Equation 1, we first implement horizontal squeeze by averaging the queried feature map along the horizontal axis. Similarly, the lower formula in Equation 1 represents vertical squeeze through vertical direction averaging. Here, *Z*^→(∗)^ denotes permuting the dimension of tensor Z as given, and *I* is an all-ones vector. The squeeze operation applied to q is simultaneously replicated on k and v, the resulting projected tensors satisfy: $q_{\left(h\right)},k_{\left(h\right)},v_{\left(h\right)}\in {\mathbb{R}}^{H\times C_{qk}},q_{\left(v\right)},k_{\left(v\right)},v_{\left(v\right)}\in {\mathbb{R}}^{W\times C_{qk}}$.

Constraining feature interactions to a single axis substantially reduces computation. The per-position output is given by Equation 2:

(2) $$y_{\left(i,j\right)}=\sum_{p=1}^{H}{\text{softmax}}_{p}(q_{(h)i}^{\text{T}}k_{(h)p})v_{(h)p}+\sum_{p=1}^{W}{\text{softmax}}_{p}(q_{(v)j}^{\text{T}}k_{(v)p})v_{(v)p}.$$

To mitigate possible contextual information loss introduced by biaxial compression, we add an auxiliary convolutional kernel to enhance local spatial details (upper part of the Attention-guide ROI Align module in **Figure 5**. Using weight matrices $W_{q}^{\left(\text{e}\right)},W_{k}^{\left(e\right)}\in {\mathbb{R}}^{C_{qk}\times C}$ and $W_{v}^{\left(\text{e}\right)}\in {\mathbb{R}}^{C_{v}\times C}$, we extract a query q_1_ from a single Anchor Line and the corresponding key *k*_1_ and value *v*_1_ from the feature map. These are concatenated along the channel dimension (with broadcasting as required) and processed by a 3×3 convolution to capture local detail. A subsequent linear projection — followed by batch normalization and an activation — reduces the concatenated channel size $\left(2C_{qk}+C_{v}\right)$ back to *C*, producing detail-enhancement weights. Finally, this enhanced feature is fused with the biaxial-attention output.

#### 2.2.3 RIoU loss

**Figure 6:**
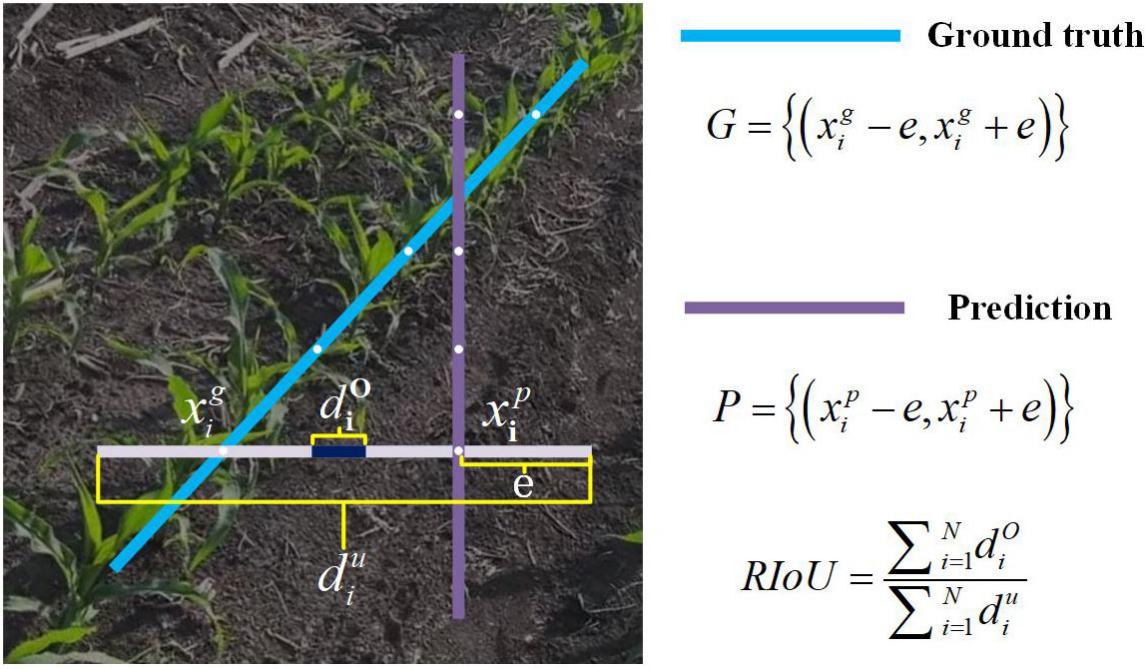
Illustration of Row IoU. Row IoU (Intersection over Union) is calculated by integrating the IoU of the extended segment at sampled positions *x_i_*.

As illustrated in **Figure 6**, each maize row is represented by a sequence of equally spaced 2D points:

(3) $$G=\{(x_{1},y_{1}),\cdots ,(x_{N},y_{N})\},$$

In Equation 3, the *y*-coordinates are sampled vertically at uniform intervals, i.e., $y_{i}=\frac{H}{N-1}\times i$, and *H* denotes the image height. Inspired by Liang et al. (2025b); Rezatofighi et al. (2019); Zheng et al. (2022), we adopt an Intersection-over-Union (IoU) based loss to measure Row IoU (RIoU), treating each maize row as an entity for regression. For each sampled position we consider the predicted point $x_{i}^{p}$ and the corresponding ground-truth point $x_{i}^{g}$, and extend both horizontally by a half-length *e*. The IoU between the two extended line segments is then:

(4) $$\text{I}oU=\frac{d_{i}^{O}}{d_{i}^{u}}=\frac{\min\;(x_{i}^{p}+e,x_{i}^{g}+e)-\max\;(x_{i}^{p}-e,x_{i}^{g}-e)}{\text{max }(x_{i}^{p}+e,x_{i}^{g}+e)-\min\;(x_{i}^{p}-e,x_{i}^{g}-e)},$$

In Equation 4, $\left[x_{i}^{p}-e,x_{i}^{p}+e\right]$ and $\left[x_{i}^{g}-e,x_{i}^{g}+e\right]$ are the extended prediction and ground-truth segments, respectively. Note that the numerator may become negative when segments do not overlap, which effectively expands the optimization space.

Treating the row as the aggregation of these sampled positions, we discretize the integral IoU and define RIoU as in Equation 5:

(5) $$RIoU=\frac{\sum_{i=1}^{N}d_{i}^{O}}{\sum_{i=1}^{N}d_{i}^{u}},$$

and the corresponding loss given in Equation 6:

(6) $$L_{RIoU}=1-RIoU,$$

where −1 ⩽ *RIoU* ⩽ 1. RIoU attains 1 when the predicted and ground-truth extended segments are perfectly aligned, and approaches -1 as they move far apart.

### 2.3 Model training

During training, following the strategy in Ge et al. (2021), one or more predicted rows are dynamically assigned as positive samples for each ground-truth maize row. The assignment cost is defined as shown in Equation 7:

(7) $$\begin{array}{c} C_{assign}=\omega_{sim}C_{sim}+\omega_{cls}C_{cls},\\ C_{sim}={\left(C_{dis}\cdot C_{xy}\cdot C_{theta}\right)}^{2}. \end{array}$$

where $C_{cls}$ denotes the focal classification cost between prediction and ground truth. The similarity cost $C_{sim}$ consists of three normalized components (all scaled to [0,1]) — $C_{dis}$ represents the average pixel distance of all valid line points, $C_{xy}$ is the distance between the start-point coordinates, and $C_{theta}$ is the angular difference θ between the predicted and ground-truth lines. The scalars $\omega_{sim}$ and $\omega_{cls}$ weight the respective costs.

The total training loss comprises classification and regression terms; regression losses are applied only to the assigned (positive) samples:

(8) $$L_{total}=\omega_{cls}L_{cls}+\omega_{xytl}L_{xytl}+\omega_{RIoU}L_{RIoU}.$$

In Equation 8, $L_{xytl}$ is the regression loss for the start-point coordinates, angle *θ* and the row length, implemented with the smooth-*l*_1_ loss. *L_xytl_* is the focal classification loss between predicted class scores and ground truth.

All experiments were performed on a machine with an Intel(R) Core(TM) i5–12500 CPU and an NVIDIA GeForce RTX 3060 (12GB) GPU. We used ResNet-18 with FPN as the backbone. The original input images (1280×720) were resized to C×W×H = 3×800×320 before training, the parameter count is 11.75M, the measured peak GPU memory usage during training was 9,878 MB. Optimization was performed with AdamW with an initial learning rate of 0.0001. A cosine decay learning-rate schedule (power factor 0.9) was used. Training ran for 70 epochs with a batch size of 40.

## 3 Results and discussion

### 3.1 Evaluation metric

We adopted the *F*1-measure as the principal evaluation metric. Intersection-over-Union (IoU) is computed between predicted rows and ground-truth rows as follows.

Predicted and ground-truth row lines are thickened to 50 pixels so that the enlarged masks closely cover the actual maize rows; IoU is then calculated between these thickened masks. A predicted row is counted as a true positive (TP) if its IoU with a ground-truth row exceeds a specified threshold. To compare localization performance more precisely across methods, we report *F*1 at multiple IoU thresholds and their mean (*mF*1):

(9) $$\begin{array}{c} F1=\frac{2\times Precision\times Recall}{Precision+Recall},\\ mF1=(F1@50+F1@55+\ldots +F1@95)/10, \end{array}$$

In Equation 9, *F*1_@_0.50*, F*1_@_0.55*,…, F*1_@_0.95 refer to the model’s *F*1 scores computed at IoU thresholds of 0.5, 0.55,…, 0.95. When the IoU threshold is 0.5, predicted corn rows are considered to reasonably represent actual corn rows in real-world scenarios. However, to precisely quantify the model’s detection accuracy and improve the ability to distinguish between different models, *F*1 scores at higher IoU thresholds are also included in the evaluation metrics.

### 3.2 Ablation study

To assess the contribution of key components in ALNet, we conducted ablation experiments on the same dataset using a ResNet-18 backbone. We progressively evaluated the RIoU loss, Feature Pyramid Network (FPN), Attention-guide ROI Align module, and DAE-Fomer. Results are summarized in **Table 1**.

**Table 1:** Effects of each component in our method.

RIoU	Attention-guide ROI align	DAE-fomer	FPN	*mF*1	*F*1@55	*F*1@65	*F*1@85	FPS
				51.90	78.37	60.32	19.54	193
✓				54.50	90.20	79.12	27.72	185.72
✓			✓	55.72	91.51	79.58	28.25	165.60
*	✓	✓	✓	56.74	90.68	79.61	31.68	163.22
✓	✓	*#*	✓	60.63	92.34	81.60	34.14	54.60
✓	✓	✓	✓	59.60	91.58	80.84	33.72	161.26

“*” Replace this module with Smooth-l1, “#” replace this module with a traditional Vision Transformer, as detailed below.

**Figure 7:**
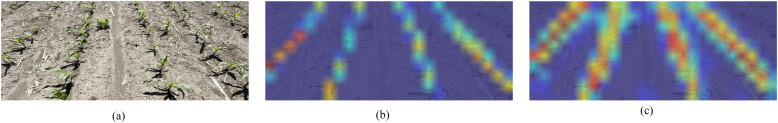
Illustration of attention weight. The red regions correspond to high score in attention weight. **(a)** represents the input image to the model; **(b)** depicts the weight heatmap of the model with smooth-*l*_1_ loss; and **(c)** depicts the weight heatmap of the model with RIoU loss.

Efficacy of the RIoU loss Incorporating the RIoU loss into the baseline model (second row in **Table 1**) increased the *mF*1 score from 51.90 to 54.50 (approximately +5%), with only a slight drop in FPS to 185.72 (less than 3%). When the RIoU loss weight was replaced by the optimal regression weight of the traditional smooth-*l*_1_ loss Yu et al. (2016), the *mF*1 score declined, confirming that RIoU loss offers greater stability and superior performance—particularly under stricter IoU thresholds such as *F*1@85 and *F*1@90. Visual comparison of weight distribution heatmaps under the two loss configurations (**Figure 7**) further validates these findings. While smooth-*l*_1_ captures the spatial representation of corn rows, the RIoU loss produces heatmaps with greater spatial smoothness and markedly higher color contrast, improving target–background separation.

This performance gain stems from RIoU’s continuous IoU-based optimization objective and favorable gradient properties. Smooth-*l*_1_, due to its linear penalty, may cause gradient oscillations when coordinate deviations are small. In contrast, RIoU yields smoother, more stable gradients and avoids stagnation when predicted and ground-truth boxes do not overlap. It also dynamically adjusts loss weights to mitigate over-sensitivity to local errors, improving convergence efficiency and representation accuracy. In summary, both quantitative results and heatmap visualizations confirm the superiority of RIoU loss in localization accuracy, gradient stability, and convergence behavior.

**Figure 8:**
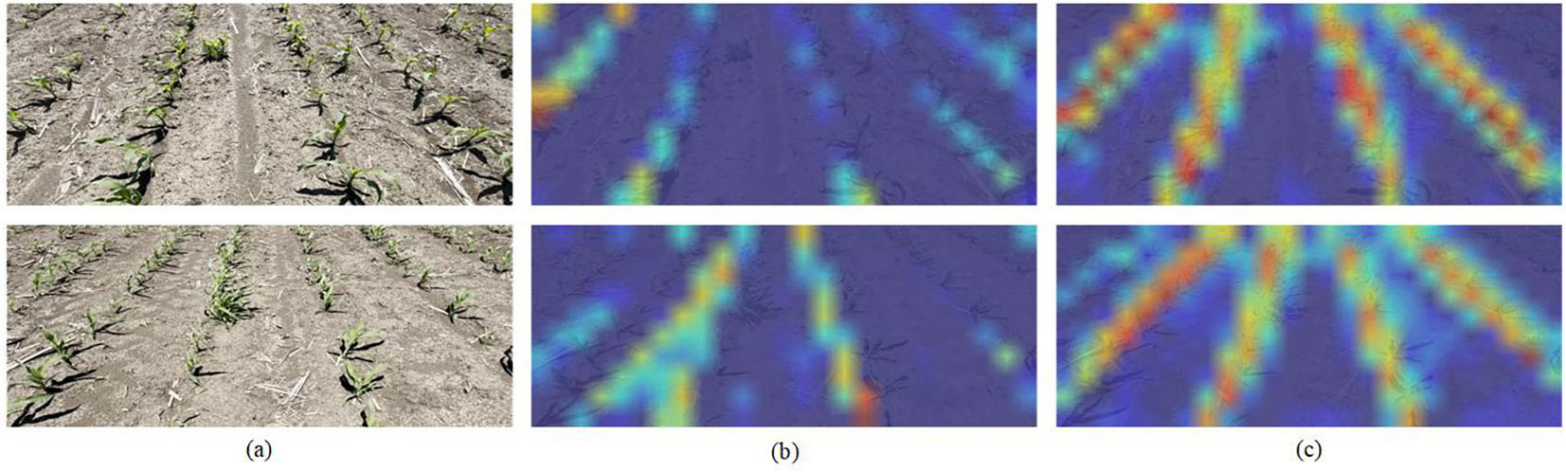
Illustration of attention weight. The red regions correspond to high score in attention weight. **(a)** represents the input image to the model; **(b)** depicts the weight heatmap of the model without FPN; and **(c)** depicts the weight heatmap of the model with FPN.

Contributions of the FPN The Feature Pyramid Network (FPN) effectively fuses deep semantic information with shallow positional cues. As **Figure 8** illustrates, attention weight heatmaps demonstrate FPN’s role in improving corn row localization accuracy and feature representation. Without FPN, heatmaps exhibit weak attention weight distributions: though centered on corn rows, response intensity is low. With FPN enabled, weights concentrate more intensely in corn row regions with heightened color saturation, indicating dual improvements in detection sensitivity and localization accuracy.

**Figure 9:**
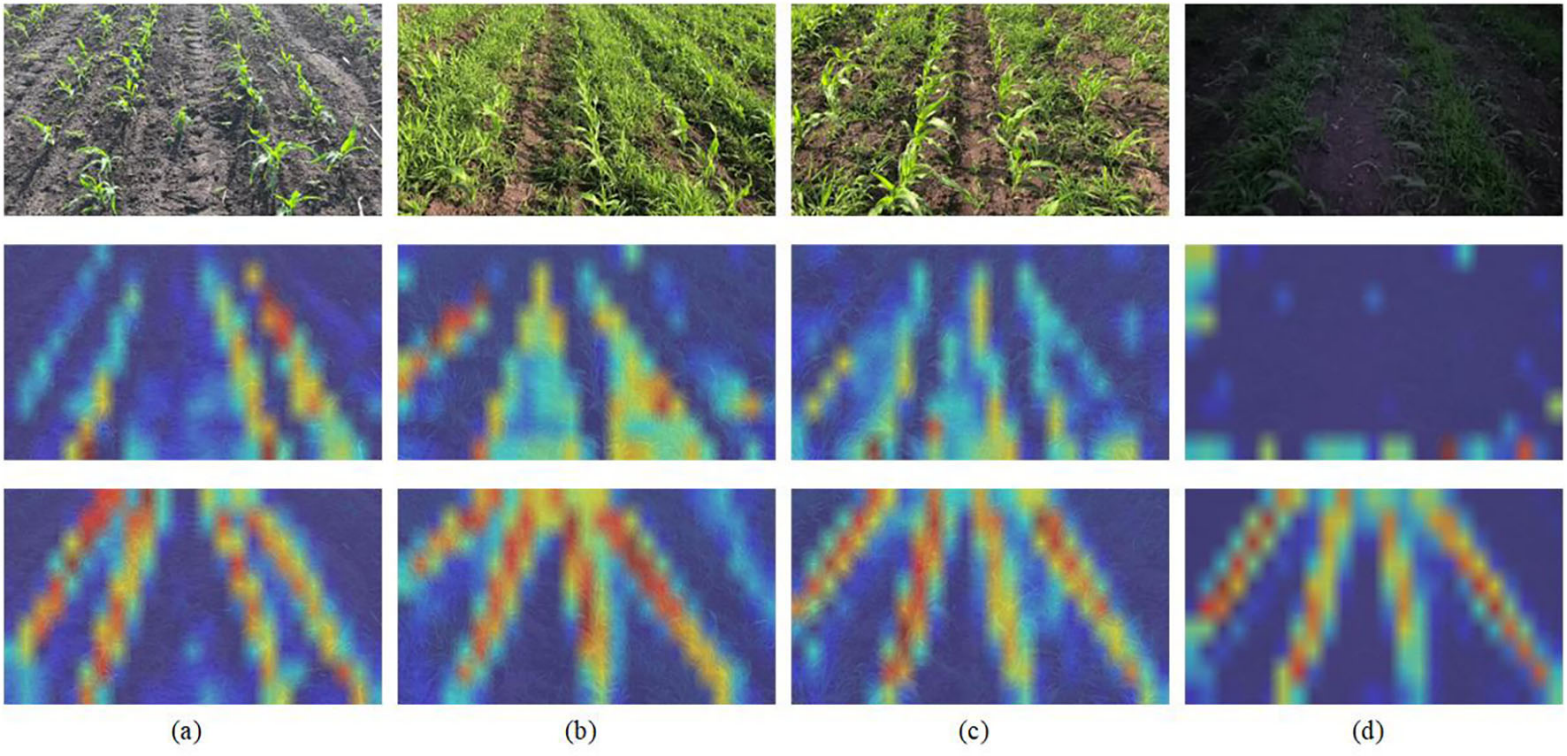
Illustration of attention weight. The red regions correspond to high score in attention weight. The first row represents the input image; the second row depicts the model without Attention-guide ROI Align; and the third row depicts the model with Attention-guide ROI Align. **(a)** depicts an ideal detection environment, while **(b, c)** depict weed-infested detection environments, and **(d)** depicts a detection environment with low-light and strong-wind conditions.

Necessity of the Attention-guide ROI Align module To validate the role of the Attention-guided ROI Align module in enhancing robustness and accuracy, we analyzed weight distribution heatmaps (**Figure 9**). As can be seen, models without this module exhibit scattered heatmap color distributions, reflecting insufficient focus on maize rows when background interference (e.g., weeds) is present. This leads to reduced feature extraction accuracy and diminished robustness under challenging conditions such as dense weeds or low light. In contrast, even under ideal conditions (clear background, sufficient lighting), the module produces more concentrated and vivid heatmap colors, confirming improved feature localization. The module provides two principal advantages: (*i*) enhanced semantic feature capture via efficient global context integration; (*ii*) achieved robust and accurate maize row localization under diverse environmental disturbances.

Effectiveness of the DAE-Fomer Replacing DAE-Former with a traditional Vision Transformer confirmed that the Dual-Axis Extrusion design achieves an optimal trade-off between detection accuracy and speed (fourth row in **Table 1**). This design overcomes the speed bottleneck of standard Transformers while maintaining high accuracy.

Summary, the ablation results demonstrate that: (*i*) RIoU loss substantially improves localization precision; (*ii*) FPN and the Attention-guided ROI Align module jointly enhance feature representation and robustness; and (*iii*) DAE-Former’s dual-axis design ensures high-speed, high-accuracy performance, validating its necessity in the corn row detection task.

### 3.3 Comparison with other methods

We compared ALNet with several mainstream detection and segmentation methods for maize-row detection. Quantitative results are reported in **Table 2**. Overall, ALNet attains a favorable trade-off between detection accuracy and inference speed: its *F*1 scores across multiple IoU thresholds exceed those of competing models, indicating a clear overall performance advantage. Further inspection of localization accuracy and real-time metrics shows that ALNet not only locates maize rows with high precision but also achieves a substantial improvement in inference speed.

**Table 2:** Performance comparison of detection models.

Method	Backbone	Parameters	mF1	F1@50	F1@70	F1@90	FPS	GFLOPs
RASCM Wei et al. (2022)	ResNet18	6.23M	39.28	72.96	46.54	6.83	200	8.4
YOLO11-N Hongbo (2024)	Darknet	3.8M	35.21	70.08	43.96	3.08	7.04	21.5
TP-ISN Chen et al. (2025)	DeepLabV3+	4.41M	50.36	81.52	62.85	7.72	46.70	19.6
DEIM-N Huang et al. (2025)	HGNetv2	4M	33.65	69.43	43.78	2.29	3.54	7
ALNet (*ours*)	ResNet18	11.23M	59.60	94.58	72.92	12.44	161.26	11.9

Specifically, relative to the semantic-segmentation method TP-ISN (DeepLabV3+), ALNet increases the average *F*1 by 9.24 percentage points while delivering a 245% improvement in FPS, demonstrating superior performance in both accuracy and speed. Compared with RASCM (ResNet-18 backbone), ALNet incurs only a 19% reduction in FPS but improves *mF*1 by 51.73 percentage points. These results indicate that ALNet more accurately regresses maize-row positions while retaining real-time capability, thus achieving a favorable balance between precision and throughput.

**Figure 10:**
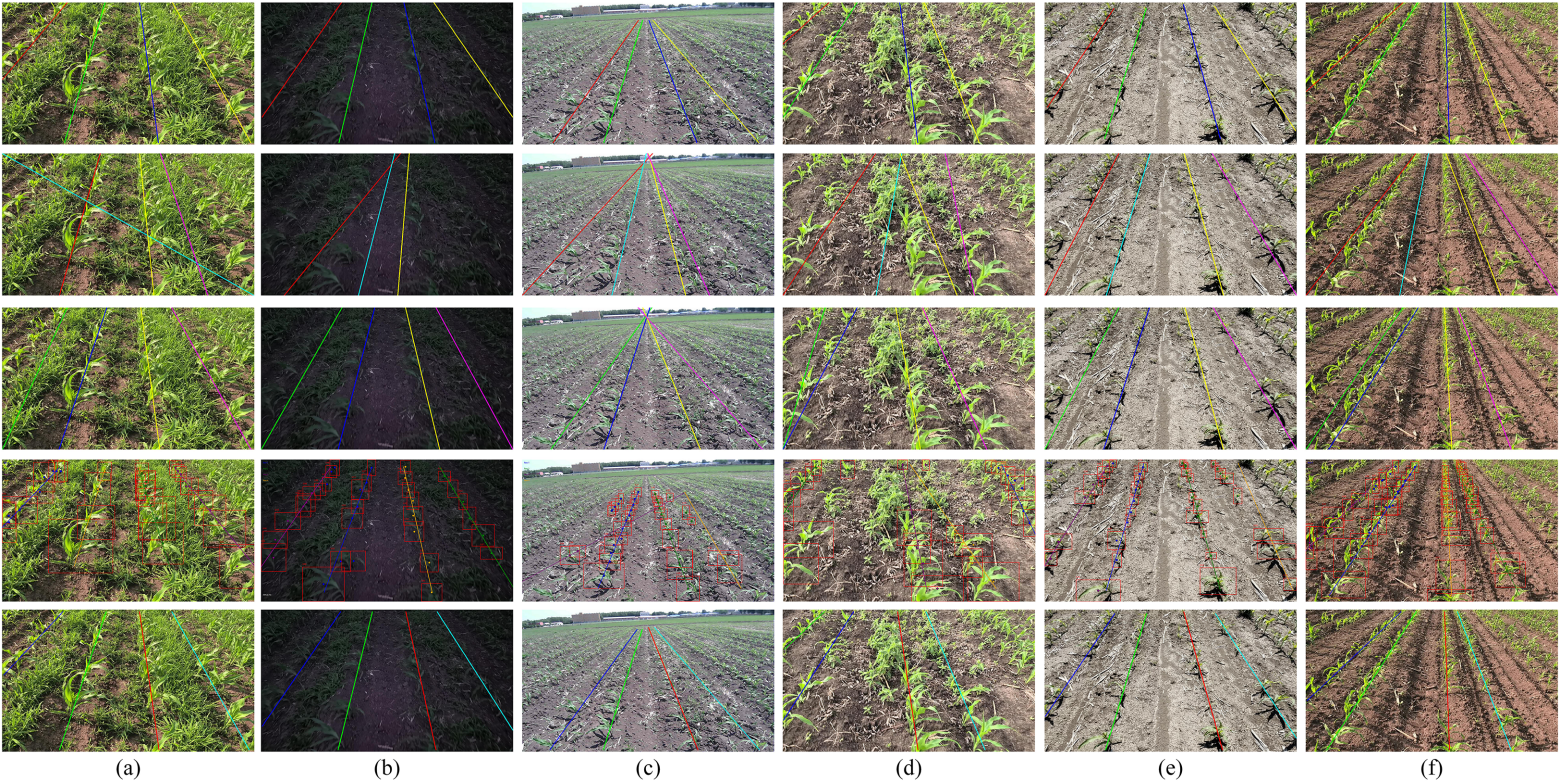
The visualization of different detection models. The first row presents the ground truth of corn rows, the second row shows the prediction maps from the RASCM model, the third row corresponds to the TP-ISN model, the fourth row displays the YOLO11 model’s predictions, and the fifth row illustrates the ALNet (ours) model’s predictions. **(a)** Weedy field with strong illumination; **(b)** low-light conditions with strong wind; **(c)** low-resolution scenario; **(d)** blurred scenario; **(e)** missing plant scenario; **(f)** overexposed illumination scenario.

**Figure 10** presents a qualitative comparison on challenging maize-row scenes. Traditional object detection frameworks, such as the YOLO series and DEIM, produce discrete bounding-box outputs that inadequately capture the continuous, strip-like geometry of maize rows. This limitation compromises geometric coherence and results in irregular edge delineation. Furthermore, the two-stage fitting approach—first detecting individual corn plants and subsequently fitting the corn seedling line—incurs substantial.

FPS degradation for these models. Instance-segmentation approaches (e.g., RASCM) attempt contour-level representation but are limited by segmentation granularity, which hinders faithful modeling of contiguous row structures. TP-ISN (DeepLabV3+), although better at preserving continuity through semantic segmentation, is constrained by its dual-branch design that relies on traditional image-processing modules (e.g., morphological operations and handcrafted post-processing) in the embedding branch; these modules limit computational efficiency and the representational capacity of learned features, thereby impairing both accuracy and real-time performance. In contrast, ALNet accurately captures the continuity and geometric characteristics of maize rows under complex conditions, producing smooth, coherent predictions that demonstrate its robustness and practical effectiveness.

**Figure 11:**
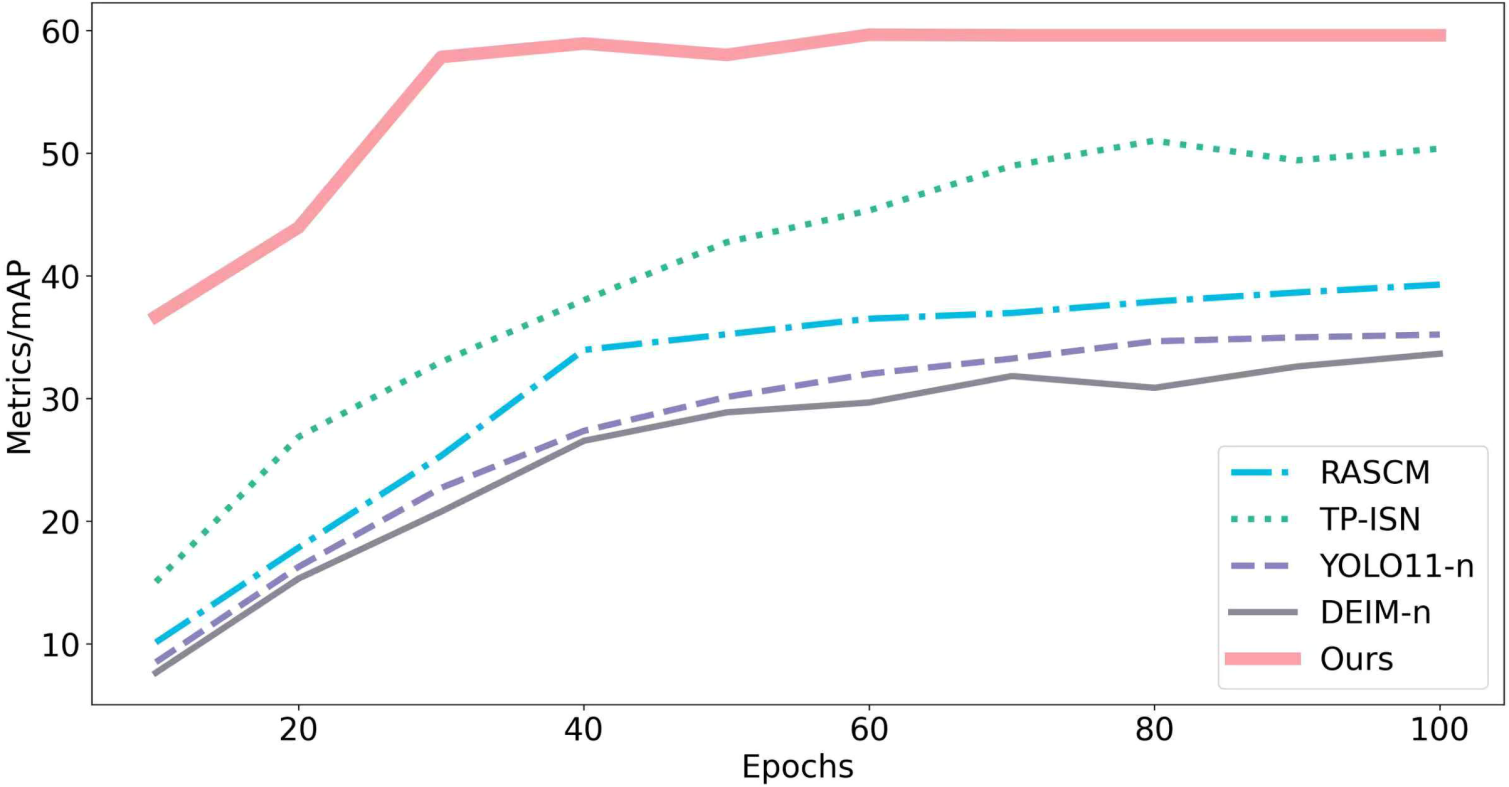
*mF*1 score *vs*. epochs for different models.

**Figure 11** shows training curves: ALNet reaches an *mF*1 of 59.60 at the seventieth training epoch, whereas competing models attain *mF*1 values in the 30–50 range under the same or larger number of epochs. This gap indicates that ALNet converges more rapidly, a consequence of the proposed architectural and optimization strategies that enable faster adaptation to the task. Under limited training resources, ALNet still achieves superior performance, validating the design philosophy of balancing training efficiency and model capability for resource-constrained deployments. In addition, the training process of ALNet is more stable and less sensitive to hyperparameters (e.g., learning rate, weight initialization), which reduces tuning effort and mitigates overfitting risk—further evidence of improved training robustness.

## 4 Limitation and future work

Our network has advanced the balance between detection accuracy and speed for maize-row detection and provides real-time, precise positional information that can support automated field-management tasks. Nevertheless, several limitations remain.

First, experiments to date have been conducted using field-collected image datasets and have not yet been validated through deployment in operational field systems. Second, owing to the seasonal growth dynamics of maize, the temporal coverage and scene diversity of the current dataset require further expansion to validate robustness across all growth stages and agricultural conditions. Thirdly, the dataset is limited to a single geographic region and lacks validation across diverse environments and corn varieties. Consequently, relying solely on this dataset may exhibit reduced detection performance or potential failure in identifying corn under different regional conditions or cultivar variations. Fourthly, while the study specifically targets corn detection during the 3–5 leaf stage, there remains a risk of performance degradation or detection failure when applied to corn at other growth stages due to the absence of cross-stage generalization validation in the current methodology.

To address these limitations, we propose the following technical directions for future work:

1. Deploy the algorithm on mobile embedded platforms to evaluate real-world inference performance and energy constraints.
2. Integrate the perception pipeline with autonomous-control modules for agricultural robots (e.g., path tracking, trajectory planning, and obstacle avoidance) to enable stable and reliable visual navigation in-field.
3. Extend training data and model generalization to additional crops with more complex planting patterns (e.g., wheat and rice) to improve multi-crop adaptability and broaden practical applicability in diverse agricultural scenarios.
4. Enrich the dataset to cover more growth stages, lighting conditions, and terrain variations, and perform field-deployment trials to validate end-to-end performance and robustness in real operational settings.

These directions will both test the practical applicability of ALNet and further enhance its generalization, efficiency, and suitability for real-world agricultural deployments.

## 5 Conclusions

This study presents ALNet (Anchor-Line Network), a lightweight convolutional neural network tailored for the elongated geometry of maize rows. By leveraging the Anchor-Line mechanism, ALNet reformulates row detection as an end-to-end regression task, avoiding the computational burden of pixel-wise prediction while preserving the holistic continuity of crop rows. The integration of an Attention-guided ROI Align module with a Dual-Axis Extrusion Transformer (DAE-Former) enables efficient global–local feature interaction, enhancing robustness under challenging field conditions such as weed infestation, low light, and wind distortion. Experimental results show that ALNet achieves a favorable accuracy–speed trade-off, with an *mF*1 of 59.60 across IoU thresholds and an inference speed of 161.26 FPS, both outperforming existing mainstream methods. Its lightweight design (11.9 GFlops) suggests potential for real-time edge deployment. These advances establish ALNet as a practical and scalable solution for visual navigation in precision agriculture.

## Data Availability

The original contributions presented in the study are included in the article/supplementary material. Further inquiries can be directed to the corresponding authors.
